# Regulatory Roles of Cytokinins and Cytokinin Signaling in Response to Potassium Deficiency in *Arabidopsis*

**DOI:** 10.1371/journal.pone.0047797

**Published:** 2012-10-24

**Authors:** Youn-Jeong Nam, Lam-Son Phan Tran, Mikiko Kojima, Hitoshi Sakakibara, Rie Nishiyama, Ryoung Shin

**Affiliations:** RIKEN Plant Science Center, Yokohama, Kanagawa, Japan; United States Department of Agriculture, Agricultural Research Service, United States of America

## Abstract

Potassium (K) is an important plant macronutrient that has various functions throughout the whole plant over its entire life span. Cytokinins (CKs) are known to regulate macronutrient homeostasis by controlling the expression of nitrate, phosphate and sulfate transporters. Although several studies have described how CKs signal deficiencies for some macronutrients, the roles of CKs in K signaling are poorly understood. CK content has been shown to decrease under K-starved conditions. Specifically, a CK-deficient mutant was more tolerant to low K than wild-type; however, a plant with an overaccumulation of CKs was more sensitive to low K. These results suggest that K deprivation alters CK metabolism, leading to a decrease in CK content. To investigate this phenomenon further, several *Arabidopsis* lines, including a CK-deficient mutant and CK receptor mutants, were analyzed in low K conditions using molecular, genetic and biochemical approaches. ROS accumulation and root hair growth in low K were also influenced by CKs. CK receptor mutants lost the responsiveness to K-deficient signaling, including ROS accumulation and root hair growth, but the CK-deficient mutant accumulated more ROS and exhibited up-regulated expression of *HAK5*, which is a high-affinity K uptake transporter gene that is rapidly induced by low K stress in ROS- and ethylene-dependent manner in response to low K. From these results, we conclude that a reduction in CK levels subsequently allows fast and effective stimulation of low K-induced ROS accumulation, root hair growth and *HAK5* expression, leading to plant adaptation to low K conditions.

## Introduction

Plants are able to grow under various nutritional environments by adapting to the conditions in which they live. If nutrients are scarce, plants regulate their metabolism through various signaling pathways in order to survive. Nutrient sensing and signaling are active throughout a plant’s life span and are important for optimal plant growth. When nutrients are limiting, plants grow at a slower rate, change their nutrient utilization and acquisition, and adjust their metabolism and morphology in order to more effectively acquire the nutrients [1], [2]. In an agricultural system, a balanced supply of soil macronutrients, especially nitrogen, phosphorus, and potassium (K), is necessary to produce the optimum quantity and quality of crops [3]. Within the plant, K is the most abundant inorganic cation, consisting of up to 1/10 of a plant’s dry weight [4]. Potassium plays various roles in the plant, such as the activation of enzymes, stabilization of protein synthesis, neutralization of negative charges on proteins, maintenance of cytoplasmic pH homeostasis [5] and osmotic balance, and the movement of other ions [3]. Potassium deprivation rapidly induces the expression of two K transporters, *HAK5,* a high-affinity K uptake transporter and *KEA5* in 6-week-old roots [6], [7], whose expression is regulated by reactive oxygen species (ROS) [7], [8]. However, while *HAK5* expression is induced at any developmental stages of roots, *KEA5* expression is not, making *HAK5* a preferable marker gene in studies of low K responses.

The relationship between the acquisition of different nutrients by mineral nutrient transporters and the imbalances triggered by a mineral deficiency are well documented [5]. For instance, nitrate transporters are down-regulated when a plant is deprived of K [9]; several nutrient transporters are up-regulated by K and phosphorus deprivation in tomato roots [10]; and when plants experience K, nitrogen, phosphorus, and sulfur deprivation, they produce ROS in roots [2], [7], [8]. Furthermore, the correlation between phytohormone signaling and nutrient signaling is well known. The K transporter TRH1 is required for root hair development and root gravitropism and functions in the auxin transporter system in *Arabidopsis* roots [11]. The genes involved in auxin biosynthesis were down-regulated by K re-supply in K-starved roots [9]. In addition, an *Arabidopsis* transcription factor, MYB77, has been shown to modulate the low K-dependent reduction of the lateral root density through auxin signal transduction [12]. Ethylene is involved in the low K signaling pathway by inducing the production of ROS in roots and then changing root hair and primary root growth and up-regulating *HAK5* expression in *Arabidopsis* [13]. Moreover, many genes respond to K starvation, which leads to increased pathogen susceptibility; a process that is linked to jasmonic acid [9].

The cytokinins (CKs) regulate various processes within plants, including cell division and root and shoot morphogenesis. In *Arabidopsis*, the key CK biosynthetic enzymes are adenosine phosphate-isopentenyltransferases (IPTs) [14]. There are two classes of IPTs in *Arabidopsis.* ATP/ADP IPTs are involved in the synthesis of *N* ^6^-(Δ^2^-isopentenyl)adenine (iP)- and *trans*-zeatin (*t*Z)-type CKs, whereas tRNA IPTs are responsible for the biosynthesis of *cis*-zeatin (*c*Z)-type CKs [14]. Additionally, it was suggested that the iP- and *t*Z-type CKs are the major forms and are more physiologically active than *c*Z-type CKs in *Arabidopsis* [15]. To exert their biological functions, CK signaling is mediated by a multi-step phosphorelay that consists of CK receptor histidine kinases (AHKs), phosphotransfer proteins (AHPs) and response regulators (ARRs). The AHKs respond to CKs by autophosphorylation and transfer of a phosphoryl group to the ARRs through the AHPs, resulting in the activation of downstream proteins [16]. Among the 8 AHKs, AHK2, AHK3 and AHK4 are implicated in CK signaling [16], [17].

It is fairly well known that interactions between nutrients and CKs influence nutrient signaling and adaptive responses in plants. Nitrate treatment induces the biosynthesis of CKs by up-regulating *IPT3* [18] and also triggers the expression of type-A *ARRs* in *Arabidopsis* [19]. CKs are also linked systemically to phosphate deprivation signaling by repressing the expression of genes that are induced by phosphate starvation conditions [20]. Through characterization of plants carrying mutations in the receptor kinases AHK3 and AHK4, it was revealed that these kinase encoding genes contribute to the repression of phosphate-starvation-responsive genes [21]. In addition, CKs were found to exert a negative effect on expression of *SULTR1;1* and *SULTR1;2*, resulting in a reduction of sulfate uptake in roots [22]. AHK3 and AHK4 are also involved in the root iron uptake machinery in *Arabidopsis* by negatively regulating the expression of genes which are induced by iron deficiency [23]. Taken together, these studies demonstrate that CKs play a role in the response to the limitations of various nutrients in plants. However, the roles of CKs in low K signaling are still unclear at the present time.

Here, we show that CK receptor mutants lose their responsiveness to low K signaling through the measurement of ROS accumulation and root growth under low K conditions. Additionally, we found that CKs affected the induction of *HAK5* expression and function under low K conditions. Finally, we provide evidence that CKs negatively regulate low K response.

## Materials and Methods

### Plant Materials and Growth Conditions

*Arabidopsis* ecotype Columbia-0 background; *IPT3*-ox [24]; the mutants *ahk2-2 (ahk2), ahk3-3 (ahk3), ahk4* (*cre1–12*), *ahk2ahk3, ahk2ahk4, ahk3ahk4* [16]; and *ipt1,3,5,7* [14] were used in our study. All seeds were sterilized and planted on normal Low Salt Medium (LSM: 1.25 mM KNO_3_, 0.5 mM KH_2_PO_4_,2 mM Ca(NO_3_)_2_, 0.75 mM MgSO_4_, 50 µM H_3_BO_3_, 10 µM MnCl, 2 µM ZnSO_4_, 1.5 µM CuSO_4_, 0.075 µM NH_4_Mo_7_O_24_, 74 µM Fe-EDTA). Four-day-old seedlings were transferred to K-sufficient LSM (+K; 0.5 M phosphoric acid, 2 mM Ca(NO_3_)_2_, 0.75 mM MgSO_4_, 2 mM Ca(NO_3_)_2_, 0.75 mM MgSO_4_, 50 µM H_3_BO_3_, 10 µ MnCl, 2 µM ZnSO_4_, 1.5 µM CuSO_4_, 0.075 µM NH_4_Mo_7_O_24_, 74 µM Fe-EDTA supplemented with 1.75 mM KCl) or K-deficient LSM (−K; 0.5 M phosphoric acid, 2 mM Ca(NO_3_)_2_, 0.75 mM MgSO_4_, 2 mM Ca(NO_3_)_2_, 0.75 mM MgSO_4_, 50 µM H_3_BO_3_, 10 µM MnCl, 2 µM ZnSO_4_, 1.5 µM CuSO_4_, 0.075 µM NH_4_Mo_7_O_24_, 74 µM Fe-EDTA supplemented with 10 µM KCl) for the growth assay as described previously [7], [13]. The K-deficient LSM used for ROS and root hair analyses did not include any additional K (0 µM KCl). To avoid any K contamination from agar, SeaKem LE Agarose (TaKaRa) was used for solidifying the LSM.

### CK Measurements

For measuring CK content, four-day-old seedlings were transferred to +K or −K LSM, and then the roots and shoots from *Arabidopsis* grown in either +K or −K conditions for 1, 3 or 7 days were harvested. More than 6 replicates per condition were analyzed. Extraction and determination of CKs were performed as previously described [25]. Statistical differences were evaluated with a *t*-test using the Graphpad Prism 5.01 software program.

### Root Assay

All seeds were planted on normal LSM and vernalized at 4°C for 3 days. Four-day-old seedlings were transferred to +K or −K medium for root assays. The plates were scanned at day 7^th^ and day 12^th^ and the images were analyzed using the Image J program. The primary root length and the number of lateral roots of more than 30 plants per treatment were measured. For the root hair analysis, two-day-old seedlings were transferred to and grown in K-sufficient and K-deficient liquid media without sucrose for 24 h. Root images were taken on a LEICA M165 FC microscope equipped with a LEICA DFC310FX camera using the Leica application suite version 3.4.1 software program. Root hair numbers were counted in a 3 mm region from the differentiation zone. To measure the length of root hairs, the longest root hairs (n = 15) in a 3 mm region from the end of the root were measured on more than 10 plants using Image J. All experiments were performed more than three times and the data presented are a representative set. Statistical differences were evaluated with One-way ANOVA and Tukey’s multiple comparison test by the Graphpad Prism 5.01 software program.

### ROS Detection Assay

To observe ROS in *Arabidopsis* roots, two-day-old seedlings were floated and grown for one day in K-sufficient and K-deficient liquid medium with or without 10 nM *t*-zeatin and without sucrose. The seedlings were treated with 20 µM of DFFDA (Invitrogen) as described previously [13]. The images of roots were taken with a LEICA M165 FC microscope equipped with a LEICA DFC310FX camera and the Leica application suite version 3.4.1 software. For ROS quantification, each image was selected within a 0.5 mm region from the starting point of the root hair differentiation zone (RHDZ). Green pixel intensity from the histogram was analyzed by the Adobe Photoshop program. The same microscopy parameters, such as exposure time, gain and contrast, were used for all repeated experiments. All ROS detection analyses were repeated more than five times. More than 15 seedlings were used for each experiment and statistical analyses were performed using the Graphpad Prism 5.01 software program. One representative set of data is shown.

### Quantitative Real-time PCR

Four-day-old seedlings were transferred and grown on K-sufficient and K-deficient media for 7 days. Roots of the seedlings were harvested in four biological samples (3 seedlings were used for each sample) for total RNA extraction using Trizol-reagent following the manufacturer’s instructions (Invitrogen). After treating with DNase I (Invitrogen), RNA was checked for genomic DNA contamination using PCR analysis. RNA (2 µg) was used as the template for reverse transcription using Superscript III (Invitrogen). Quantitative real-time PCR was performed using the THUNDERBIRD SYBR qPCR mix (Toyobo) and the MX3000P PCR machine (Agilent Technologies). An actin gene (AT3G18780) was used as a control and amplified with sense (5′-CTGGATCGGTGGTTCCATTC-3′) and antisense (5′-CCTGGACCTGCCTCATCATAC-3′) primers. For analyzing *HAK5* gene (AT4G13420) expression, a sense primer (5′-CGAGACGGACAAAGAAGAGGAACC-3′) and antisense primer (5′-CACGACCCTTCCCGACCTAATCT-3′) were used. MX3000P version 4 (Agilent Technologies) was used for calculating and analyzing the threshold cycle (Ct) values. The fold change of target transcript compared with the K-sufficient-grown wild-type (WT) control was calculated by normalization with the Actin Ct values. The expression level of Actin was not affected by K starvation. Statistical differences in target gene transcripts were evaluated by One-way ANOVA using ΔΔCt values [26].

## Results

### Low K Status *in planta* Results in Reduction of CK Content

**Figure 1:**
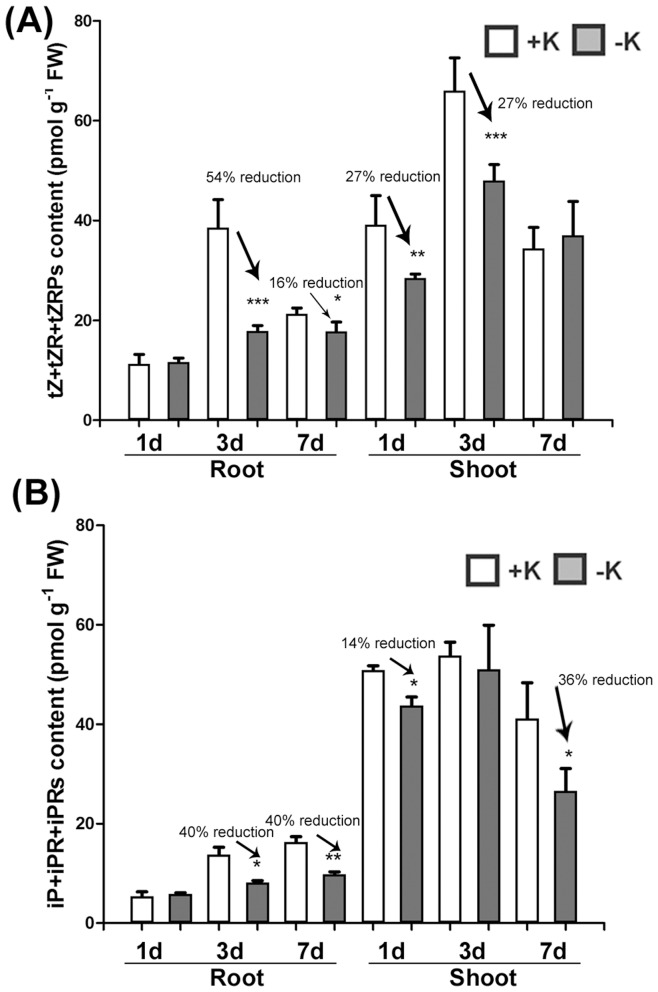
K deprivation reduces CK content. Analysis of CK content in roots and shoots treated with K-sufficient (+K) or K-deficient (−K) conditions for one, three or seven days. (A) The content of *t*Z-type (*t*Z + *t*ZR + *t*ZRPs) CKs. (B) The content of iP-type (iP + iPR + iPRPs). White bar indicates CK content in K-sufficient grown plants and gray bar indicates CK content in K-deficient grown plants. Each error bar indicates standard error and * indicates the statistical difference between +K and −K (*P<0.05,**P<0.01; Student *t*-test) (n>6).

To investigate whether CK metabolism is affected by low K status, the content of CKs in K-deficient and K-sufficient-grown *Arabidopsis* WT Col-0 shoots and roots were analyzed in a time-course manner (Figure 1 and Table S1). Most samples contained undetectable amounts of the dihydrozeatin-type (DZ) CKs. The contents of *cis*-zeatin (*c*Z), *cis*-zeatin riboside (*c*ZR), and *cis*-zeantin riboside phosphates (*c*ZRPs) were not significantly altered by low K (Table S1). Among the bioactive CKs, the levels of the *t*Z-type (Figure 1A) and iP-type CKs (Figure 1B) clearly decreased in the roots of plants grown on media with lower K for three or seven days. In shoot tissues, *t*Z-type CKs were mainly reduced in plants grown on media with lower K for one or three days (Figure 1A); and iP-type CKs were reduced in plants grown on lower K media for one or seven days (Figure 1B). These results demonstrate that CK content is negatively affected by low K conditions.

### The CK-deficient *ipt1,3,5,7* Mutant Enhanced Root Growth while the CK-overaccumulating *IPT3*-ox Suppressed Root Growth Under Low K Conditions

**Figure 2:**
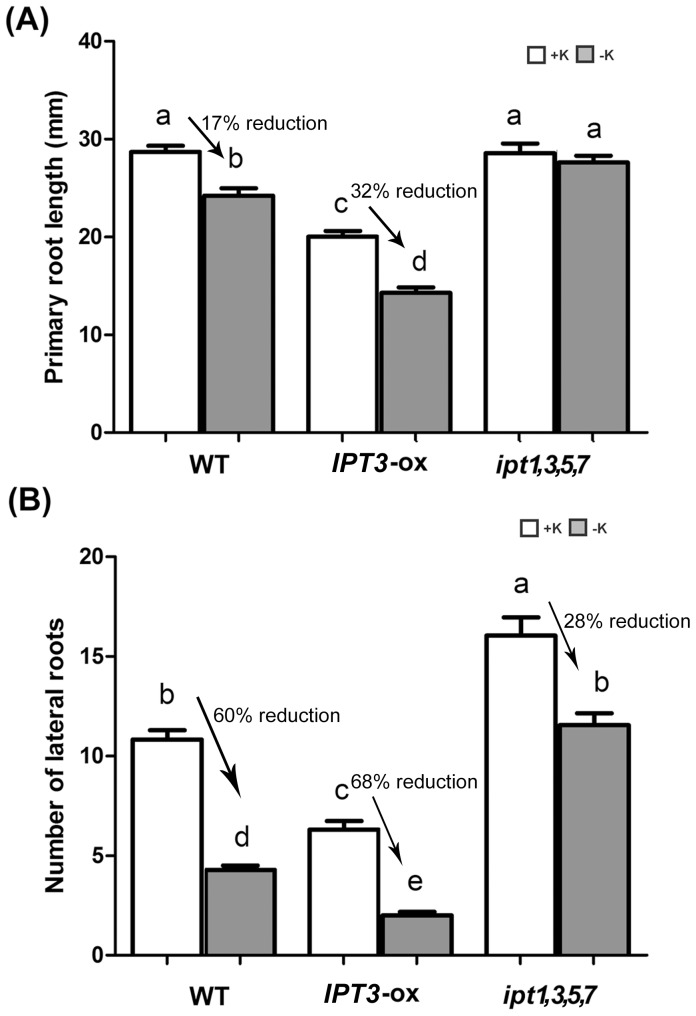
Root growth assay of *Arabidopsis* WT, *IPT3*-ox and *ipt1,3,5,7* plants under +K and -K conditions. Plants were grown under +K conditions for 4 days and then transferred and grown on +K or −K medium for 7 days. Length of primary root (A) and number of lateral roots (B) were analyzed (n>30). Different letters indicate significant differences from each other as determined using ANOVA(P<0.05) and significances were corrected post hoc using Tukey’s HSD comparisons.

The *ipt1,3,5,7* line carries mutations in ATP/ADP isopentenyltransferases, leading to markedly reduced bioactive endogenous *t*Z-type and iP-type CKs [14]. On the other hand, the transgenic plant overexpressing *IPT3* (*IPT3*-ox) highly accumulates CKs in relative comparison to WT plants [24]. Primary root growth and lateral root numbers were analyzed in *ipt1,3,5,7* and *IPT3*-ox plants under both K-sufficient (1.75 mM KCl) and K-deficient (10 µM KCl) conditions in order to understand whether low K signaling is affected by the level of endogenous CKs. To optimize the nutrient composition for *Arabidopsis* and to modify K content, LSM was used for all experiments [7], [8], [13]. All seedlings were germinated on full nutrient LSM and at four days were transferred to medium containing 1.75 mM KCl (+K) or 10 µM KCl (–K). When *ipt1,3,5,7*, *IPT3*-ox and WT plants were grown under K-deficient conditions for 7 days and compared with K-sufficient-grown plants, the primary root length of *IPT3*-ox plants (32% reduction) showed 15% more reduction compared to WT (17% reduction) but *ipt1,3,5,7* roots showed no significant changes (Figure 2A). Twelve-day-old K-deficient-grown WT showed a 36% reduction of primary root growth compared to K-sufficient-grown WT. On the other hand, *IPT3*-ox plants showed a 54% reduction and *ipt1,3,5,7* only showed a 20% decrease in root growth (Figure S1B). Since *ipt1,3,5,7* is not a complete CK-null mutant, even though there was no difference in root growth for 7 day K-sufficient-grown and K-deficient-grown *ipt1,3,5,7,* longer K-deficient treatment (12 days) showed mild reduction in root growth (Figure S1B). The discrepancy between the data observed with 7- and 12-day-low K treated plants might be attributed to the age-dependent expression of the ATP/ADP IPT6 encoding gene. This gene weakly expresses in young seedlings, and its expression is increased with the aging of the plants [27]. In addition, K-deficient-grown *ipt1,3,5,7* had a 38% reduction in lateral root numbers compared to K-sufficient-grown *ipt1,3,5,7*. However, WT had a 61% decrease in lateral root numbers responding to K deficiency and *IPT3*-ox had a 68% decrease (Figure 2B). These results suggest that CKs negatively influence root growth rate and an increase in endogenous CK content results in reduced tolerance to K deficiency.

### Loss of Root Growth Response to K Deprivation in *ahk* Mutants

**Figure 3:**
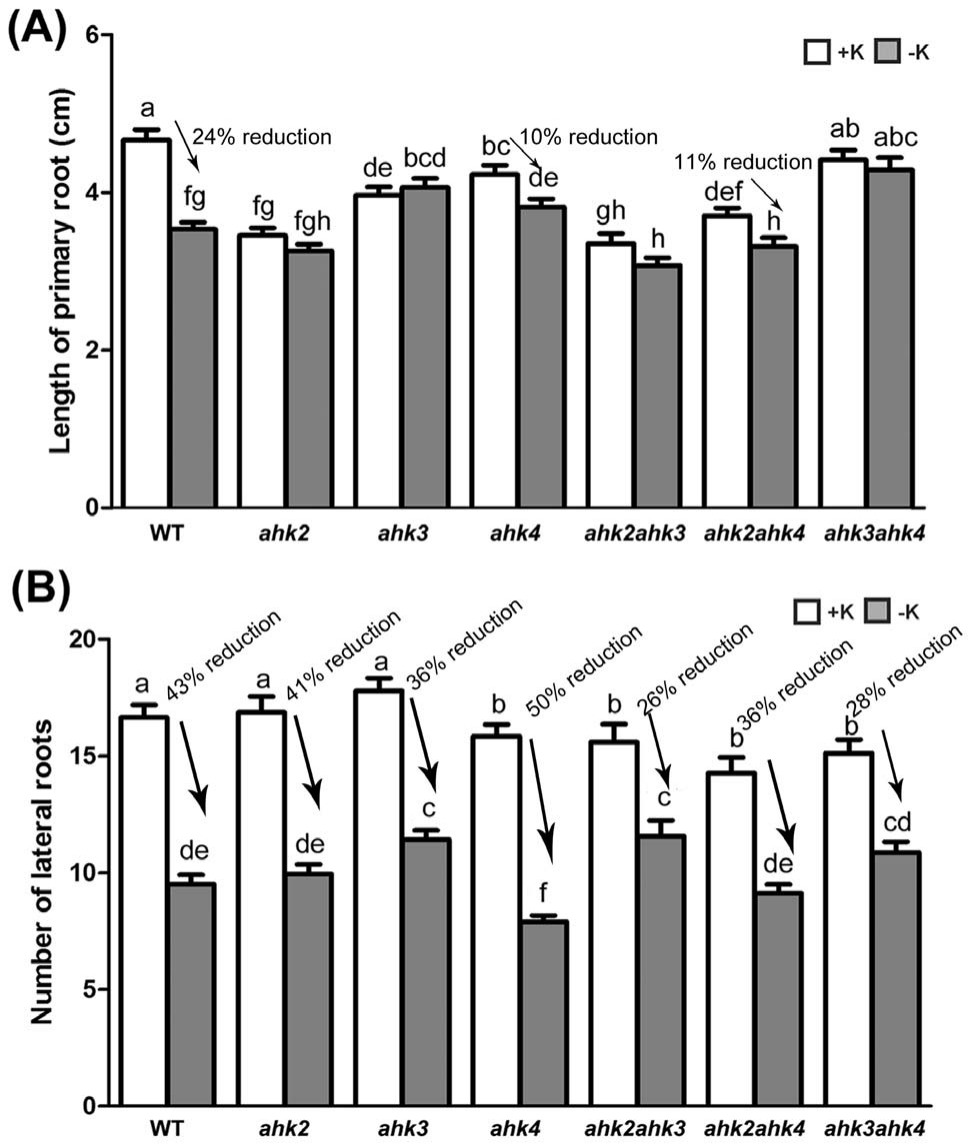
Root growth assay of WT and *ahk* mutants under +K and −K conditions. Plants were grown under +K conditions for 4 days and then transferred and grown on +K or −K medium for 7 days. Length of primary root (A) and number of lateral roots (B) were analyzed (n>30). Different letters indicate significant differences from each other as determined using ANOVA (P<0.05) and significances were corrected post hoc using Tukey’s HSD comparisons.

To further understand the role of CK signaling under low K conditions, primary root length and lateral root numbers of the WT, *ahk* single mutants (*ahk2*, *ahk3* and *ahk4*) and *ahk* double mutants (*ahk2ahk3, ahk2ahk4* and *ahk3ahk4*) were analyzed. Statistically significant differences of P<0.05 among WT *ahk* single and double mutants were determined using ANOVA; and significances were corrected post hoc using Tukey’s HSD comparisons. Unlike WT plants, which exhibited decreased primary root length under K-starved conditions, the primary root growth of both *ahk* single (except *ahk4*) and *ahk* double mutants was not affected by the −K conditions (Figure 3A). While lateral root numbers in WT and *ahk* single mutants exhibited similar responses to low K conditions (Figure 3B), the *ahk* double mutants, especially *ahk2ahk3*, showed a reduction in responsiveness of lateral root growth under the same conditions (Figure 3B). These results suggested that the repression of primary root growth, and to some extent the lateral root growth, by K starvation was mediated by CK signaling, especially through AHK2 and AHK3.

### CKs Function in Low K-dependent ROS Accumulation

**Figure 4:**
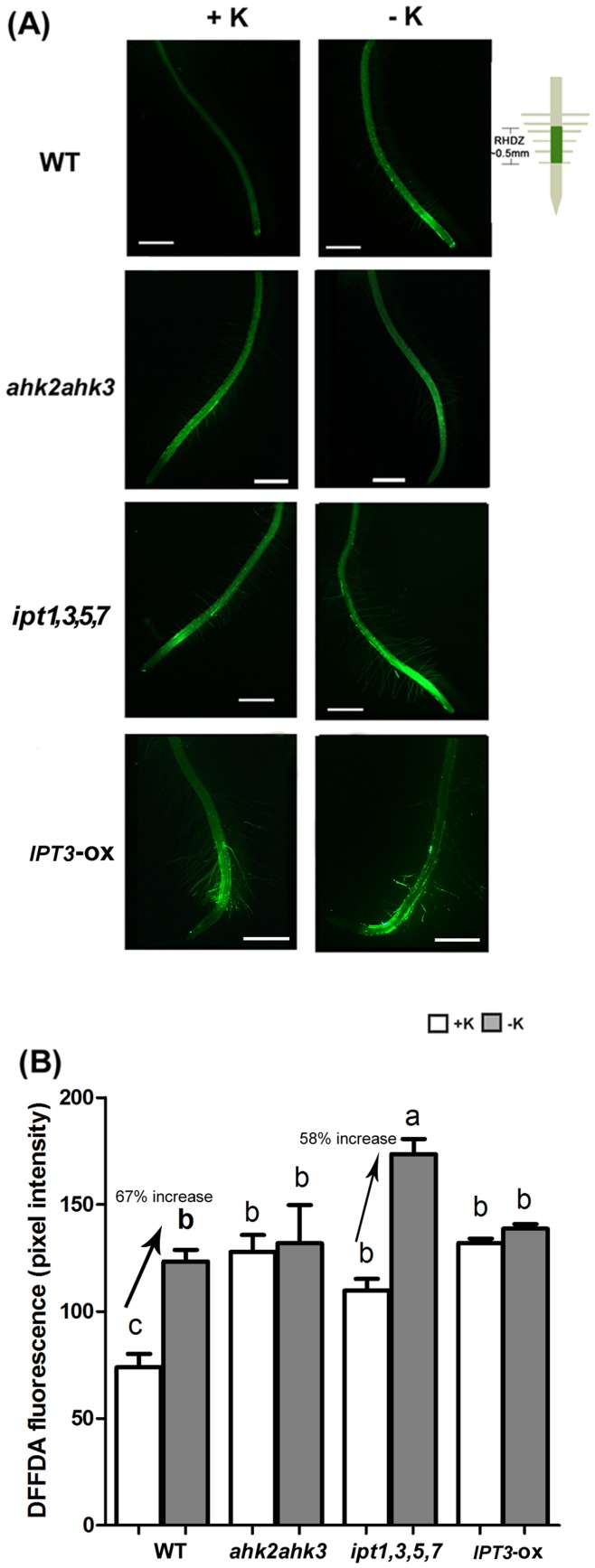
Low K-dependent ROS accumulation was obstructed in *ahk2ahk3* mutant and enhanced in *ipt1,3,5,7* mutant. (A) Pseudo-colored ROS fluorescence signals were detected in WT, *ahk2ahk3* and *ipt1,3,5,7* roots after staining with 20 µM of DFFDA for 20 min. Bar indicates 0.5 mm. (B) Quantification of DFFDA fluorescence signal shown in Figure 4A. Pixel intensity of the roots was measured from the root hair differentiation zone to 0.5 mm. Different letters indicate significant differences from each other as determined using ANOVA (P<0.05) and significances were corrected post hoc using Tukey’s HSD comparisons. (n>20).

Previous studies have demonstrated that low K conditions induce ROS accumulation in *Arabidopsis* roots [7], [13]. To examine whether CK signaling is involved in the accumulation of ROS under low K conditions, ROS accumulation in RHDZ of WT, *ahk2ahk3* and *ipt1,3,5,7* was analyzed under +K and −K conditions using the membrane-permeable fluorescent dye 5-(and 6-) carboxyl-2′,7′-difluorodihydrofluorescein diacetate (DFFDA; Figure 4A). After obtaining images, the signal intensity within a region 0.5 mm from the starting point of the RHDZ was calculated (Figure 4B). Under K-sufficient conditions, there was greater accumulation of ROS in the RHDZ of *ahk2ahk3* relative to WT. Similar to what we observed with primary root growth, ROS accumulation was not increased in the *ahk2ahk3* mutant under K-deficient conditions. This finding suggests that AHK2- and AHK3-dependent CK signaling is required for low K-dependent ROS accumulation (Figure 4). Similar to the result of *ahk2ahk3*, higher ROS levels were detected in *ipt1,3,5,7* and *IPT3*-ox RHDZ than that of WT under +K conditions. We did not observe significant change in ROS level in K-deficient grown *IPT3*-ox plants. However, a significant increase in ROS level was noted for K-deficient grown *ipt1,3,5,7* (Figure 4). The enhanced ROS production under K deficiency conditions in plants with low level of CKs supports the hypothesis that low CK levels are associated with enhanced low K stress tolerance, which is also consistent with the observed reduction of CK content under K-deficient conditions (Figure 1).

### CKs Influence Root Hair Development Under K-deficient and K-sufficient Conditions

**Figure 5:**
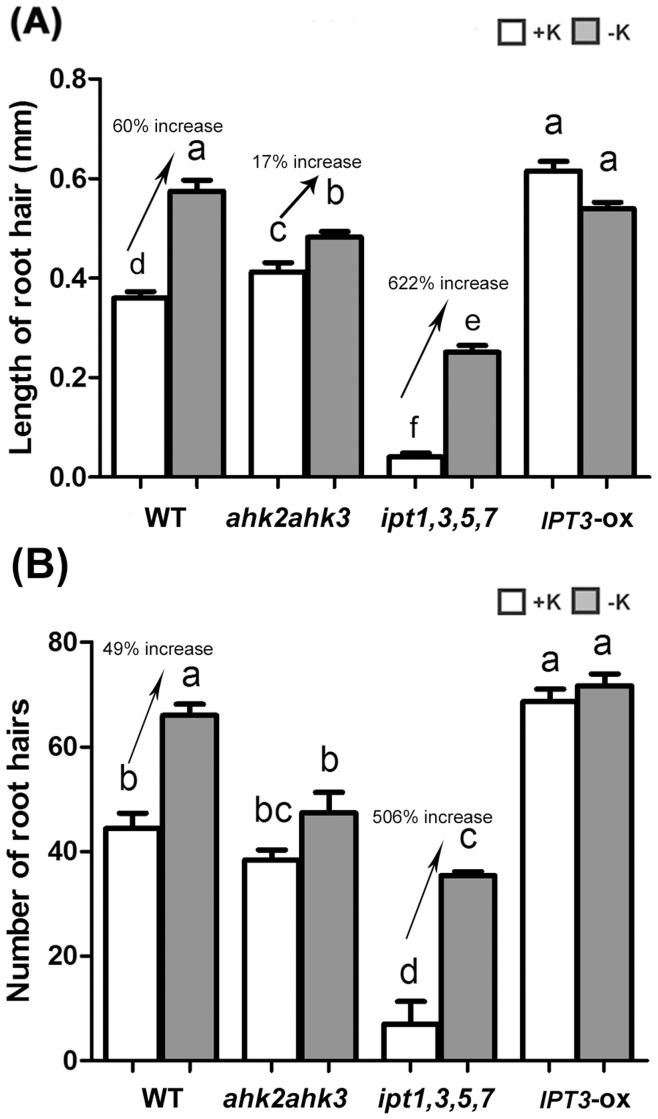
Root hair growth analysis in WT, *ahk2ahk3* and *ipt1,3,5,7* plants. Root hair length (A) and number of root hairs (B) in the seedlings were analyzed. The number of root hairs was counted in a 3 mm region from the starting point of the RHDZ. In order to measure root hair length, the longest root hairs (n = 8) per seedling (n>10) were chosen and measured. Different letters indicate significant differences from each other as determined using ANOVA (P<0.05) and significances were corrected post hoc using Tukey’s HSD comparisons.

ROS is known to be an essential signal for root hair elongation [28]. Induction of root hair elongation by low K requires ethylene-dependent ROS accumulation [13]. In order to determine whether CKs exert influence on the low K-dependent induction of root hair development, root hair growth in the WT, *ahk2ahk3, ipt1,3,5,7* and *IPT3*-ox plants was analyzed (Figure 5). As previously reported, the root hairs of K-deficient WT plants were much longer than those of K-sufficient WT plants (Figure 5) [13]. In *ahk2ahk3* and *IPT3*-ox, root hair length was longer than that in WT under K-sufficient conditions, but the induction degree of root hair length in the *ahk2ahk3* (17% increase) and the *IPT3*-ox (no significant change) by low K treatment was much lower than in WT (60%) (Figure 5A). K-sufficient-grown *ipt1,3,5,7* seedlings have much shorter root hairs than WT and *ahk2ahk3,* but K-deficiency led to a dramatic induction in root hair growth of *ipt1,3,5,7* in comparison with WT (Figure 5A). Similar results were obtained from analyses of root hair numbers in the RHDZ responding to low K conditions (Figure 5B). K-sufficient-grown *ipt1,3,5,7* had fewer root hairs than WT, *ahk2ahk3* and *IPT3*-ox but K-deficient-grown *ipt1,3,5,7* dramatically increased root hair numbers. However, the numbers of root hairs in *ahk2ahk3* and *IPT3*-ox were not affected by K status (Figure 5B). These data indicated that the degree of CK reduction was important for the induction of root hair elongation to cope with low K conditions. Similar to ROS accumulation, the induction of root hair growth by K deprivation is also negatively correlated with endogenous CK content and modulated via AHK2/AHK3-meditated CK signaling.

### CKs Alter K-deficient Induced *HAK5* Expression

A high-affinity *Arabidopsis* K transporter, HAK5, is one of the key proteins functioning in low K signaling and is transcriptionally regulated by K limitation [29]. To investigate whether CKs regulate low K-induced gene expression, *HAK5* expression was analyzed by real-time PCR in the CK receptor mutant, *ahk2ahk3*, the CK-overaccumulating *IPT3*-ox line, and the CK-deficient *ipt1,3,5,7* mutant under K-sufficient and K-deficient conditions (Table 1). Under K-sufficient conditions, the expression level of *HAK5* was lower in the *ahk2ahk3* mutant and remarkably higher in *IPT3*-ox than in WT plants. However, *HAK5* expression under insufficient K remained unchanged in the *ahk2ahk3* mutant. Interestingly, the induction of *HAK5* expression by K deficiency was greatly suppressed in *IPT3*-ox but highly activated in *ipt1,3,5,7* compared to WT (Table 1). These results indicate that the expression of *HAK5* under low K conditions is regulated by both CK-dependent and CK-independent mechanisms and CKs negatively regulate *HAK5* gene expression in response to K starvation.

**Table 1:** Relative *HAK5* expression levels in WT and in *ahk2ahk3, IPT3*-ox and *ipt1,3,5,7* under +K and -K conditions.

	Fold Change compared to WT +K	
	+K	−K
WT	1.00±0^b^	157.81±18.55^e^
*ahk2ahk3*	0.85±0.10^a^	156.73±24.39^e^
IPT3-ox	3.21±1.09^c^	17.68±3.49^d^
*ipt1,3,5,7*	1.03±0.09^b^	559.72±61.05^f^

The fold changes of *HAK5* expression were compared to K-sufficient-grown WT. The actin gene was used as internal control. Data represent the means and SE values of four independent biological replicates. Different letters indicate the statistically different datasets from One-way ANOVA. Experiments were repeated four times using four different biological samples. Each biological sample was harvested from three different plants.

## Discussion

In this report, we describe the functional analyses of CKs and CK-related signaling in response to K deficiency by investigating the consequences of altered CK contents and the suppression of CK signaling. Results from both gain- and loss-of-function studies suggest that CKs may function as negative regulators in response to low K conditions (Figure 2 and 3). CK content was decreased in low-K-grown roots and shoots (Figure 1). In addition, the induction level of the *HAK5* gene by low K was decreased in *IPT3*-ox plants (Table 1). Consistent with this result, the expression of *HAK5* was more highly induced by low K conditions in the CK-deficient *ipt1,3,5,7* mutant as compared to WT (Table 1). Moreover, the growth of both primary and lateral roots in *IPT3*-ox plants under low K conditions was more highly suppressed than in WT (Figure 2). Collectively, these data strongly indicate the negative regulatory roles of CKs metabolism in primary and lateral root growth responding to K deficiency.

Previous studies have shown that multiple phytohormones regulate low-K signaling, that can lead to effects on gene expression, reduced primary root growth, reduced lateral root growth, and increased root hair growth [7], [9], [12], [13], [30]. One typical phenotype of low-K-grown *Arabidopsis* plants is the reduction of lateral root numbers. Auxin is known to be a positive regulator of lateral root development [31]. Low K-dependent reductions of lateral root development are known to be regulated by auxin via reductions of auxin levels and transport. Independent from primary and lateral root growth, ethylene has been reported to act as a positive regulator of low K-dependent ROS accumulation, *HAK5* expression and the induction of root hair growth [13]. Low K also leads to a decrease in primary root growth [29] that may be regulated by ABA [30]. Jasmonate was also shown to regulate low K-dependent gene expression [9]. In this study, we found that CKs function differently from other hormones by acting as negative regulators in low K-dependent *HAK5* expression (Table 1), primary root growth (Figure 2A and 3A) and root hair growth (Figure 5). These data suggest that CKs might function in parallel with ethylene but in an antagonistic behavior in low K signaling.

CKs are known to exert influence on the acquisition of several macronutrients, such as nitrogen, phosphorus and sulfur. Specifically, the expression of genes encoding multiple macronutrient transporters, including nitrate transporters, sulfate transporters and phosphate transporters, were decreased by CKs [32]. In the case of nitrogen, CKs and nitrogen are reciprocally influenced. CK content was tightly regulated by nitrogen supply. Higher nitrate-grown *Arabidopsis* had higher CK levels than low nitrate-grown *Arabidopsis*. In addition, CKs act as long distance root-to-shoot signals and local signals for nitrate sensing [33]. CKs could negatively regulate nitrogen uptake via the control of nitrate and ammonium transporter gene expression [32], [34]. Other phosphate and sulfate transporters were regulated similar to nitrate transporters [20], [22]. In our study, we also showed that CKs negatively regulate the gene expression of the high-affinity K transporter *HAK5* (Table 1). Moreover, the levels of bioactive CKs were reduced in both roots and shoots with the most drastic reduction observed in roots after 3 days of K deprivation (Figure 1A). Collectively, our results support that CKs function as negative regulators of *HAK5* gene expression; a regulation that is similar to that of other macronutrient transporters.

In this study, we have also demonstrated that CKs control the response to low K conditions through CK signaling by functional analyses of the *ahk* mutants in response to K deficiency. The results of root growth assays indicated that among the three CK receptor kinases, AHK2 and AHK3 play major roles in the regulation of the response to K deficiency (Figure 3). The weak correlation between AHK4 and low K signaling may be explained by its dual activity. In the presence of CKs, AHK4 possesses kinase activity and phosphorylates AHPs; however, in the absence of CKs, AHK4 acts as a phosphatase that dephosphorylates AHPs [35]. This finding differs from the regulation of other macronutrients by CKs. AHK3- and/or AHK4-dependent CK signaling was proposed to have dominant roles in the function of nitrate, phosphate and sulfate transporters [16], [21], [22], [23], [36]. These data suggest that there might be some specificity of CK signaling to each macronutrient signaling pathway and that AHK2 and AHK3 might have major roles in low K signaling.

As a common response to K deficiency, ROS is induced in roots, leading to root hair elongation [2], [7], [8], [13]. The investigation of ROS induction in *ahk2ahk3* roots further supports the observation that CK signaling is involved in the response to low K. Results shown in Figure 4 indicated that the ROS accumulation was not altered in the *ahk2ahk3* mutant by K availability, whereas a significant difference was observed in ROS accumulation in WT either with or without K. Similar to low K-dependent primary root growth, we only observed a slight responsiveness of root hair elongation to low K in the *ahk2ahk3* mutant (Figure 5). Consistent with these data, several ethylene insensitive mutants, including *ein2-1*, *etr1-1,* and *etr1-3*, showed a smaller increase of ROS in low K-grown roots. However, these ethylene insensitive mutants still show the low K-dependent ROS accumulation in roots [13], suggesting that other ethylene receptors or unidentified ethylene receptors regulate low K signaling. Our data indicates that AHK2 and AHK3 are the major CK receptors for low K-dependent ROS accumulation.

In *ipt1,3,5,7* mutant, shorter and fewer root hairs were found under K-sufficient conditions compared to WT. This phenomenon may be due to the lower endogenous CKs content and is not related to K responsiveness. However, the increase in root hair ratio of *ipt1,3,5,7* in response to low K is more than 10 times higher than that of WT (Figure 5). Reduction ratio of CKs responding to low K compared to +K seems to be more important than the absolute value of CKs for root hair growth. In addition, the *ipt1,3,5,7* has lower active CK levels in +K conditions. Even though actual decreased CK content responding to low K may be lower in *ipt1,3,5,7*, plants showed more sensitive response to low K. Because *ipt1,3,5,7* has lower CK level, the threshold value for root hair growth responding to low K may be much lower than WT. These data suggest that the CK deficient *ipt1,3,5,7* has lower response threshold level to K deficiency-induced root hair growth.

It is well known that CKs and ABA have antagonistic effects on responses to a number of stresses, including drought and high salinity [37], [38], [39], [40], [41]. Our data, together with previously published results, demonstrate that CKs and ABA also have opposite effects on the response to low K conditions as well [30]. In accordance with their function, CK levels were decreased in WT plants under K-deficient conditions (Figure 1), whereas ABA levels increased [30].

**Figure 6:**
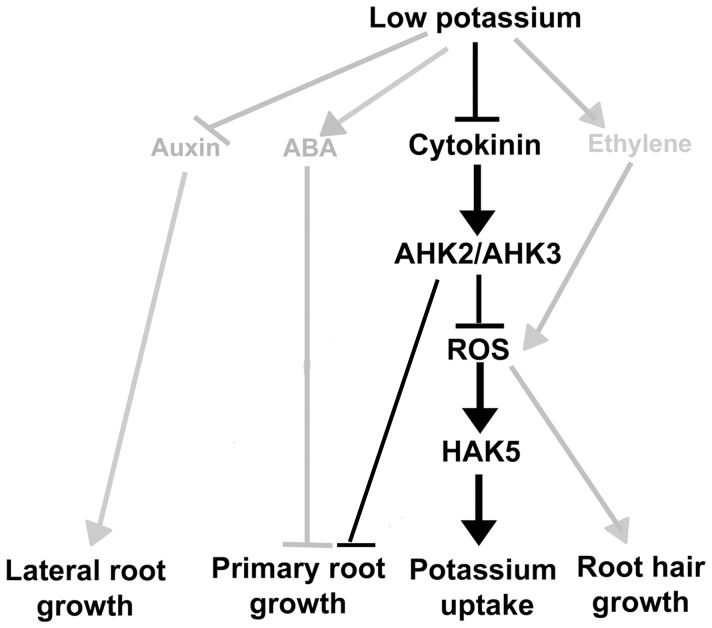
A schematic model for the roles of CKs in low K signal pathway. Low K conditions lead to reduced levels of endogenous CKs, which are negative regulators under these conditions. Lower CK levels result in ROS production, *HAK5* expression, and then altered root growth. Previous studies showed that ethylene is a positive regulator in low K signaling that controls ROS production, *HAK5* expression as well as root hair growth [13]. ABA [30] and auxin [12] also are involved in controlling low K signaling pathway. Arrowhead line, positive regulation; bar-head lines, negative regulation; gray color, previously identified; black color, identified in this study.

Taken together, we propose a model in which plants adapt to K deprivation by triggering an efficient regulation of various hormonal biosynthesis and signal cascades, including CKs and CK signaling (Figure 6) [7], [12], [13], [30]. Specifically, for CKs and CK signaling, we suggest that during K deprivation, CK metabolism is altered, leading to a decrease in CKs. A reduction in CK levels subsequently allows fast and effective stimulation of low K-induced ROS accumulation, root hair growth and *HAK5* expression, leading to plant adaptation to low K conditions.

## Supporting Information

Figure S1 **Root growth assay of WT,** ***IPT3*** **-ox,** ***ipt1,3,5,7*** **and** ***ahk2ahk3*** **under +K and -K conditions.** (A) WT, *IPT3*-ox, *ipt1,3,5,7* and *ahk2ahk3* grown under +K and −K conditions for 7 days. (B) Root growth assay of WT, *IPT3-ox* and *ipt1,3,5,7* plants under +K and −K conditions for 12 days. Plants were grown under +K conditions for 4 days and then transferred and grown on +K or −K medium for 12 days. Length of primary roots were analyzed (n>30). Significant differences were represented by different letters on the bars (P<0.05; *t*-test).
(JPG)

Table S1 **CK content in K-deficient- and K-sufficient-grown WT plants.** Measurement of CK content in roots and shoots under both +K and -K conditions. *t*Z, *trans*-zeatin; *t*ZR, *t*Z riboside; *c*Z, *cis*-zeatin; *c*ZR, *c*Z riboside; iP, *N* ^6^-(Δ^2^-isopentenyl)adenine; iPR, iP riboside; DZ, dihydrozeatin; DZR, DZ riboside; *t*Z7G, *t*Z-7-*N*-glucoside; *t*Z9G, *t*Z-9-*N*-glucoside; *t*ZOG, *t*Z-*O*-glucoside; *t*ZROG, *t*ZR-*O*-glucoside; *c*ZOG, *c*Z-*O*-glucoside; *c*ZROG, cZR-*O*-glucoside; DZ9G, DZ-9*-N*-glucoside; iP7G, iP-7-*N*-glucoside; iP9G, iP-9 *-N* -glucoside; *t*ZRPs, *t*ZR phosphates; *c*ZRPs, *c*ZR phosphates; iPRPs, iPR phosphates; DZRPs, DZR phosphates; *c*ZRPOG, *c*ZR phosphate-*O*-glucoside; *t*ZRPOG, *t*ZR phosphate-*O-*glucoside (n>6).
(XLS)

## References

[1] Lopez-Bucio J, Cruz-Ramirez A, Herrera-Estrella L (2003) The role of nutrient availability in regulating root architecture. Curr Opin Plant Biol 6: 280–287. 12753979 10.1016/s1369-5266(03)00035-9

[2] Schachtman DP, Shin R (2007) Nutrient sensing and signaling: NPKS. Annu Rev Plant Biol 58: 47–69. 17067284 10.1146/annurev.arplant.58.032806.103750

[3] Amtmann J, Amtmann K (2006) Strength training for EMS professionals: fit to respond. J Emergency Med Services 31: 52–56. 10.1016/S0197-2510(06)70448-X 16818113

[4] Leigh RA, Jones RGW (1984) A hypothesis relating critical potassium concentrations for growth to the distribution and functions of this ion in the plant cell. New phytol 97: 1–13.

[5] Marschner H (1995) Minera nutrient of higher plants. Academic Press, London 2nd Edn.

[6] Gierth M, Maser P, Schroeder JI (2005) The potassium transporter AtHAK5 functions in K^(+)^ deprivation-induced high-affinity K^(+)^ uptake and AKT1 K^(+)^ channel contribution to K^(+)^ uptake kinetics in *Arabidopsis* roots. Plant Physiol 137: 1105–1114. 15734909 10.1104/pp.104.057216 PMC1065410

[7] Shin R, Schachtman DP (2004) Hydrogen peroxide mediates plant root cell response to nutrient deprivation. Proc Natl Acad Sci USA 101: 8827–8832. 15173595 10.1073/pnas.0401707101 PMC423280

[8] Shin R, Berg RH, Schachtman DP (2005) Reactive oxygen species and root hairs in *Arabidopsis* root response to nitrogen, phosphorus and potassium deficiency. Plant Cell Physiol 46: 1350–1357. 15946982 10.1093/pcp/pci145

[9] Armengaud P, Breitling R, Amtmann A (2004) The potassium-dependent transcriptome of *Arabidopsis* reveals a prominent role of jasmonic acid in nutrient signaling. Plant Physiol 136: 2556–2576. 15347784 10.1104/pp.104.046482 PMC523322

[10] Wang YH, Garvin DF, Kochian LV (2002) Rapid induction of regulatory and transporter genes in response to phosphorus, potassium, and iron deficiencies in tomato roots. Evidence for cross talk and root/rhizosphere-mediated signals. Plant Physiol 130: 1361–1370. 12428001 10.1104/pp.008854 PMC166655

[11] Vicente-Agullo F, Rigas S, Desbrosses G, Dolan L, Hatzopoulos P, et al (2004) Potassium carrier TRH1 is required for auxin transport in *Arabidopsis* roots. Plant J 40: 523–535. 15500468 10.1111/j.1365-313X.2004.02230.x

[12] Shin R, Burch AY, Huppert KA, Tiwari SB, Murphy AS, et al (2007) The *Arabidopsis* transcription factor MYB77 modulates auxin signal transduction. Plant Cell 19: 2440–2453. 17675404 10.1105/tpc.107.050963 PMC2002618

[13] Jung JY, Shin R, Schachtman DP (2009) Ethylene mediates response and tolerance to potassium deprivation in *Arabidopsis* . Plant Cell 21: 607–621. 19190240 10.1105/tpc.108.063099 PMC2660615

[14] Miyawaki K, Tarkowski P, Matsumoto-Kitano M, Kato T, Sato S, et al (2006) Roles of Arabidopsis ATP/ADP isopentenyltransferases and tRNA isopentenyltransferases in cytokinin biosynthesis. Proc Natl Acad Sci USA 103: 16598–16603. 17062755 10.1073/pnas.0603522103 PMC1637627

[15] Sakakibara H (2006) Cytokinins: activity, biosynthesis, and translocation. Annu Rev Plant Biol 57: 431–449. 16669769 10.1146/annurev.arplant.57.032905.105231

[16] Higuchi M, Pischke MS, Mahonen AP, Miyawaki K, Hashimoto Y, et al (2004) In planta functions of the *Arabidopsis* cytokinin receptor family. Proc Natl Acad Sci USA 101: 8821–8826. 15166290 10.1073/pnas.0402887101 PMC423279

[17] Nishimura C, Ohashi Y, Sato S, Kato T, Tabata S, et al (2004) Histidine kinase homologs that act as cytokinin receptors possess overlapping functions in the regulation of shoot and root growth in *Arabidopsis* . Plant Cell 16: 1365–1377. 15155880 10.1105/tpc.021477 PMC490032

[18] Takei K, Ueda N, Aoki K, Kuromori T, Hirayama T, et al (2004) AtIPT3 is a key determinant of nitrate-dependent cytokinin biosynthesis in Arabidopsis. Plant Cell Physiol 45: 1053–1062. 15356331 10.1093/pcp/pch119

[19] Scheible WR, Morcuende R, Czechowski T, Fritz C, Osuna D, et al (2004) Genome-wide reprogramming of primary and secondary metabolism, protein synthesis, cellular growth processes, and the regulatory infrastructure of *Arabidopsis* in response to nitrogen. Plant Physiol 136: 2483–2499. 15375205 10.1104/pp.104.047019 PMC523316

[20] Martin AC, del Pozo JC, Iglesias J, Rubio V, Solano R, et al (2000) Influence of cytokinins on the expression of phosphate starvation responsive genes in *Arabidopsis* . Plant J 24: 559–567. 11123795 10.1046/j.1365-313x.2000.00893.x

[21] Franco-Zorrilla JM, Martin AC, Leyva A, Par-Ares JP (2005) Interaction between phosphate-starvation, sugar, and cytokinin signaling in *Arabidopsis* and the roles of cytokinin receptors CRE1/AHK4 and AHK3. Plant Physiol 138: 847–857. 15923327 10.1104/pp.105.060517 PMC1150402

[22] Maruyama-Nakashita A, Nakamura Y, Yamaya T, Takahashi H (2004) A novel regulatory pathway of sulfate uptake in *Arabidopsis* roots: implication of CRE1/WOL/AHK4-mediated cytokinin-dependent regulation. Plant J 38: 779–789. 15144379 10.1111/j.1365-313X.2004.02079.x

[23] Seguela M, Briat JF, Vert G, Curie C (2008) Cytokinins negatively regulate the root iron uptake machinery in *Arabidopsis* through a growth-dependent pathway. Plant J 55: 289–300. 18397377 10.1111/j.1365-313X.2008.03502.x

[24] Galichet A, Hoyerova K, Kaminek M, Gruissem W (2008) Farnesylation directs AtIPT3 subcellular localization and modulates cytokinin biosynthesis in *Arabidopsis* . Plant Physiol 146: 1155–1164. 18184738 10.1104/pp.107.107425 PMC2259095

[25] Kojima M, Kamada-Nobusada T, Komatsu H, Takei K, Kuroha T, et al (2009) Highly sensitive and high-throughput analysis of plant hormones using MS-probe modification and liquid chromatography-tandem mass spectrometry: an application for hormone profiling in *Oryza sativa* . Plant Cell Physiol 50: 1201–1214. 19369275 10.1093/pcp/pcp057 PMC2709547

[26] Yuan JS, Reed A, Chen F, Stewart CN Jr (2006) Statistical analysis of real-time PCR data. BMC Bioinformatics 7: 85. 16504059 10.1186/1471-2105-7-85 PMC1395339

[27] Miyawaki K, Matsumoto-Kitano M, Kakimoto T (2004) Expression of cytokinin biosynthetic isopentenyltransferase genes in Arabidopsis: tissue specificity and regulation by auxin, cytokinin, and nitrate. Plant J 37: 128–138. 14675438 10.1046/j.1365-313x.2003.01945.x

[28] Foreman J, Demidchik V, Bothwell JH, Mylona P, Miedema H, et al (2003) Reactive oxygen species produced by NADPH oxidase regulate plant cell growth. Nature 422: 442–446. 12660786 10.1038/nature01485

[29] Qi Z, Hampton CR, Shin R, Barkla BJ, White PJ, et al (2008) The high affinity K^+^ transporter AtHAK5 plays a physiological role in planta at very low K^+^ concentrations and provides a caesium uptake pathway in *Arabidopsis* . J Exp Bot 59: 595–607. 18281719 10.1093/jxb/erm330

[30] Kim MJ, Shin R, Schachtman DP (2009) A nuclear factor regulates abscisic acid responses in *Arabidopsis* . Plant Physiol 151: 1433–1445. 19759343 10.1104/pp.109.144766 PMC2773093

[31] Reed RC, Brady SR, Muday GK (1998) Inhibition of auxin movement from the shoot into the root inhibits lateral root development in *Arabidopsis* . Plant Physiol 118: 1369–1378. 9847111 10.1104/pp.118.4.1369 PMC34753

[32] Brenner WG, Romanov GA, Kollmer I, Burkle L, Schmulling T (2005) Immediate-early and delayed cytokinin response genes of *Arabidopsis thaliana* identified by genome-wide expression profiling reveal novel cytokinin-sensitive processes and suggest cytokinin action through transcriptional cascades. Plant J 44: 314–333. 16212609 10.1111/j.1365-313X.2005.02530.x

[33] Sakakibara H, Takei K, Hirose N (2006) Interactions between nitrogen and cytokinin in the regulation of metabolism and development. Trends Plant Sci 11: 440–448. 16899391 10.1016/j.tplants.2006.07.004

[34] Kiba T, Naitou T, Koizumi N, Yamashino T, Sakakibara H, et al (2005) Combinatorial microarray analysis revealing *Arabidopsis* genes implicated in cytokinin responses through the His->Asp Phosphorelay circuitry. Plant Cell Physiol 46: 339–355. 15695462 10.1093/pcp/pci033

[35] Mahonen AP, Bishopp A, Higuchi M, Nieminen KM, Kinoshita K, et al (2006) Cytokinin signaling and its inhibitor AHP6 regulate cell fate during vascular development. Science 311: 94–98. 16400151 10.1126/science.1118875

[36] To JP, Haberer G, Ferreira FJ, Deruere J, Mason MG, et al (2004) Type-A *Arabidopsis r*esponse regulators are partially redundant negative regulators of cytokinin signaling. Plant Cell 16: 658–671. 14973166 10.1105/tpc.018978 PMC385279

[37] Chow B, McCourt P (2004) Hormone signalling from a developmental context. J Exp Bot 55: 247–251. 14673027 10.1093/jxb/erh032

[38] Hansen H, Dörffling K (2003) Root-derived trans-zeatin riboside and abscisic acid in drought-stressed and rewatered sunflower plants: interaction in the control of leaf diffusive resistance? Funct Plant Biol 30: 363–375. 10.1071/FP02223 32689021

[39] Pospisilova J, Vagner M, Malbeck J, Travnickova A, Batkova P (2005) Interactions between abscisic acid and cytokinins during water stress and subsequent rehydration. Biol Plantarum 49: 533–540.

[40] Ha S, Vankova R, Yamaguchi-Shinozaki K, Shinozaki K, Tran LS (2012) Cytokinins: metabolism and function in plant adaptation to environmental stresses. Trends Plant Sci. 17: 172–179. 10.1016/j.tplants.2011.12.005 22236698

[41] Nishiyama R, Watanabe Y, Fujita Y, Le DT, Kojima M, et al (2011) Analysis of cytokinin mutants and regulation of cytokinin metabolic genes reveals important regulatory roles of cytokinins in drought, salt and abscisic acid responses, and abscisic acid biosynthesis. Plant Cell. 23: 2169–2183. 10.1105/tpc.111.087395 PMC3160038 21719693

