# Supplementary figures and images for: Regulatory Roles of Cytokinins and Cytokinin Signaling in Response to Potassium Deficiency in Arabidopsis
Source: PLoS One. 2012 Oct 24;7(10):e47797. doi: 10.1371/journal.pone.0047797 (PMC3480408; doi:10.1371/journal.pone.0047797)

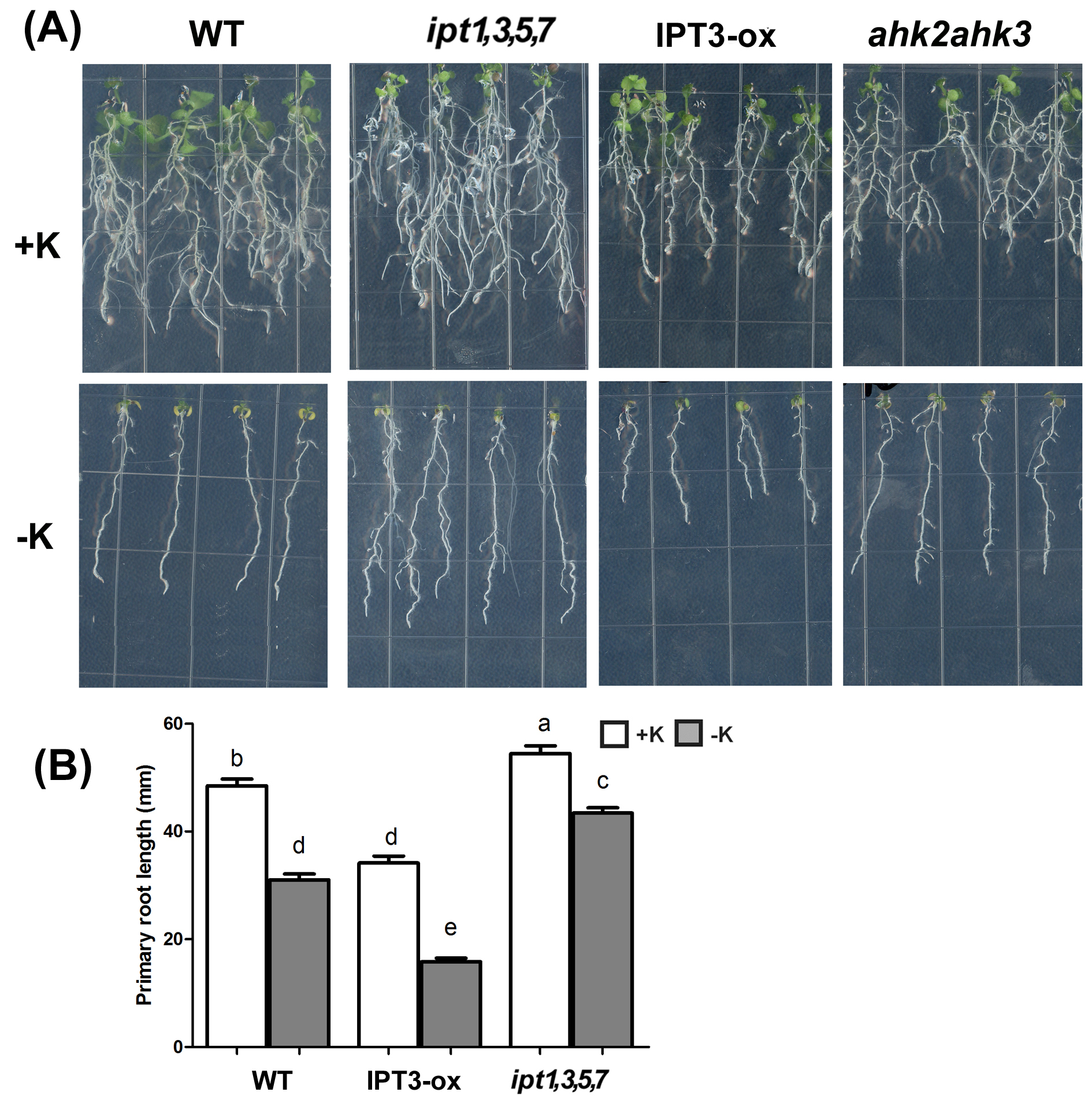

Supplement: Figure S1 — Root growth assay of WT, IPT3 -ox, ipt1,3,5,7 and ahk2ahk3 under +K and -K conditions. (A) WT, IPT3-ox, ipt1,3,5,7 and ahk2ahk3 grown under +K and −K conditions for 7 days. (B) Root growth assay of WT, IPT3-ox and ipt1,3,5,7 plants under +K and −K conditions for 12 days. Plants were grown under +K conditions for 4 days and then transferred and grown on +K or −K medium for 12 days. Length of primary roots were analyzed (n>30). Significant differences were represented by different letters on the bars (P<0.05; t-test). (JPG) [file pone.0047797.s001.jpg]
